# Research on the establishment of a four-classification model for breast mass ultrasound images based on transfer learning

**DOI:** 10.1371/journal.pone.0340111

**Published:** 2026-01-05

**Authors:** Jinlao Li, Bo Zhao, YongQi Chen, Guang Wang, XueLiang Tian, ShuYu Zhou

**Affiliations:** 1 Diagnostic Ultrasound Center, The First Hospital of Jilin University, Changchun, China; 2 Breast Surgery Department, The First Hospital of Jilin University, Changchun, China; 3 Pharmaceutical College, University Of beihua University, Yanji, China; King Abdulaziz University, SAUDI ARABIA

## Abstract

Breast mass classification via ultrasound is critical for early diagnosis but remains challenging due to overlapping morphological features. This study evaluates the efficacy of optimized transfer learning (TL) based on deep convolutional neural networks (DCNNs) in distinguishing four breast mass categories: invasive ductal carcinoma (IDC), fibroadenoma (FA), mucinous carcinoma (MC), and inflammatory mass (IM). Our approach comprises four key steps: (1) applying extensive data augmentation to a retrospective dataset of 346 ultrasound images from 294 patients (November 2021-March 2023) classified by pathological confirmation; (2) implementing transfer learning with five pretrained DCNN models (ResNet18, ResNet50, DenseNet121, MobileNetV2, GoogLeNet); (3) optimizing model hyperparameters for four-class classification; (4) comparing performance metrics (accuracy, precision, recall, F1-score, AUC-ROC) against two senior radiologists (8- and 10-years’ experience) on a held-out test set. Results: The DenseNet121 and GoogLeNet models demonstrated superior overall accuracy (0.912 and 0.926, respectively), significantly outperforming the radiologists’ consensus (0.691) (p < 0.01). In class-specific analysis, the optimized models achieved notably higher accuracy for IDC, MC, and IM. Statistical testing confirmed the significant performance improvement of the top models over human experts. Optimized TL with DCNNs, particularly DenseNet121 and GoogLeNet, enables highly accurate four-class breast mass classification in ultrasound images, surpassing expert radiologist performance with statistical significance. This approach holds promise for clinical decision support. Code is publicly available at https://github.com/jinlao777/BCC.

## 1. Introduction

In recent years, breast diseases have become one of the most common medical issues among women in China. According to the report from the American Cancer Society [1], The incidence and mortality rates of breast cancer remain persistently high each year, posing a serious threat to women’s health. Research has found that early screening and diagnosis are crucial for the treatment and prognosis of breast cancer. Common imaging methods include mammography, ultrasound, and magnetic resonance imaging [2]. Among them, breast ultrasound examination has attracted much attention due to its advantages of non-ionizing radiation, rapid imaging, portability, and low cost. In clinical practice, radiologists classify ultrasound images as benign or malignant based solely on experience and provide preliminary diagnostic results. This diagnostic process is subjective and highly dependent on the experience of the radiologist. Therefore, since the 1980s, computer-aided diagnosis (CAD) systems have been used to assist radiologists in improving the efficiency of medical image diagnosis, and have achieved good results [3].

Machine learning is a core area of computer algorithms that can automatically improve through experience, making it a key application area of artificial intelligence. Common machine learning algorithms include random forests, support vector machines, and neural networks. Deep learning is a branch of machine learning built on neural network algorithms and is therefore also known as deep convolutional neural networks (DCNNs) [4]. Deep learning is a widely recognized method capable of processing raw image data and automatically learning high-level abstract features from it. This gives it the potential to perform tasks in medical image analysis, including nodule classification and organ segmentation [5], This greatly reduces the time required for manual feature extraction in traditional machine learning processes, and it is also capable of handling large-scale data samples. Furthermore, it is a machine learning method based on multi-layer neural network structures, which processes information and makes predictions by automatically learning and adjusting the connection strengths between different layers [6].

Currently, deep learning-related methods have become the mainstream technology in the field of computer-aided diagnosis, widely applied to various tasks, including disease classification, Region of Interest (ROI) segmentation, object detection, and image registration [7], The applications of deep learning are also widespread [5], including but not limited to the breast [8], liver [9], carotid artery [10], thyroid [11], and lungs [12].

However, research on deep learning in the field of breast ultrasound image classification often focuses on the classification of benign and malignant nodules. There is limited research and application regarding the rich information present in breast mass ultrasound images for further detailed classification [13–17]. Therefore, expanding the scope of research on breast ultrasound image classification to focus more attention on detailed classification of breast masses is of significant importance for improving diagnostic accuracy and clinical applicability. This study deliberately selected four categories that present significant challenges and hold considerable importance in clinical diagnosis. Research has found that fibroadenomas can increase the subsequent risk of breast cancer by approximately 2 to 3 times [18]. Furthermore, although fibroadenomas typically exhibit sonographic features of benign masses on ultrasound, many present with atypical characteristics in clinical practice. These atypical features include irregular shape, heterogeneous internal echogenicity, indistinct or irregular margins, angulations, or microlobulations, and may also exhibit rich blood flow signals, creating a significant overlap in ultrasound appearance with breast carcinoma.

Mucinous carcinoma of the breast, conversely, is a unique pathological type of breast cancer that occurs predominantly in postmenopausal or elderly patients [19]. Its histological presentation is distinctive, often showing low cellular atypia. On ultrasound images, the masses frequently demonstrate a relatively regular shape and well-defined borders, with complex internal echogenicity. These characteristics often lead to misdiagnosis as fibroadenomas or other benign lesions, thereby increasing the risk of a missed diagnosis [20].

Inflammatory breast masses, as a common benign breast condition, have a relatively low incidence. Particularly for non-lactational inflammatory masses, the exact etiology remains unclear. Their clinical manifestations and physical findings can resemble infectious mastitis or inflammatory carcinoma. In most cases, a multidisciplinary approach is required to narrow the differential diagnosis [21]. Sonographically, some of their features bear a high resemblance to breast cancer and may be confused with it. This not only poses challenges for early diagnosis but also may cause patients to miss the optimal treatment window, thereby presenting a significant challenge to hospital diagnostic capabilities [22].

Invasive ductal carcinoma (IDC) is the most common type of breast cancer clinically, typically developing when ductal cancer cells breach the basement membrane and invade the stroma [23]. Its microscopic features include cancer cells with considerable variation in size and shape, marked pleomorphism, frequent mitotic figures, arranged in cord-like or nest-like structures, sometimes accompanied by glandular formations. Characteristic ultrasound findings include a small, hard mass with marked posterior acoustic shadowing; a spiculated or crab-leg-like margin is its most distinctive sonographic feature [24]. However, these subtle features may be insufficiently pronounced or overlooked on ultrasound, particularly in some early-stage cases, potentially leading to inadequate attention during the diagnostic process.Here, we present the first systematic evaluation of optimized transfer learning for four-category classification of breast ultrasound masses (invasive ductal carcinoma, fibroadenoma, mucinous carcinoma, and inflammatory masses), demonstrating superior diagnostic accuracy to senior radiologists through rigorous benchmarking of five DCNN architectures.

Our principal contributions are threefold:(1)We propose a comprehensive transfer learning framework that systematically optimizes and benchmarks five contemporary DCNN architectures (ResNet18, ResNet50, DenseNet121, MobileNetV2, GoogLeNet) for the challenging task of four-category breast mass classification. (2) We establish a rigorous clinical benchmark by comparing model performance directly against two senior radiologists with 8- and 10-years of experience, providing a realistic assessment of clinical applicability. (3)We demonstrate that our optimized DenseNet121 model not only achieves state-of-the-art classification accuracy but also significantly outperforms human experts across all four mass categories, offering a potent tool for clinical decision support. (4)To ensure full reproducibility and foster further research, we will make our complete code implementation publicly available on GitHub.

The remainder of this paper is organized as follows. Section 2 details the methodology, including data collection procedures, preprocessing techniques, deep learning model architectures, and statistical analysis. Section 3 presents the experimental results, comprising patient characteristics and ultrasound image features, along with performance comparisons between transfer learning models and radiologists. Section 4 provides a comprehensive discussion of the findings, clinical implications, study limitations, and future research directions.The paper concludes with Section 5, which summarizes the principal findings and their clinical significance.

## 2. Materials and methods

This retrospective study was approved by the Ethics Committee of the First Bethune Hospital of Jilin University, and written informed consent was waived (Approval No. 2023-KS-191).

### 2.1 Data collection

**Fig 1:**
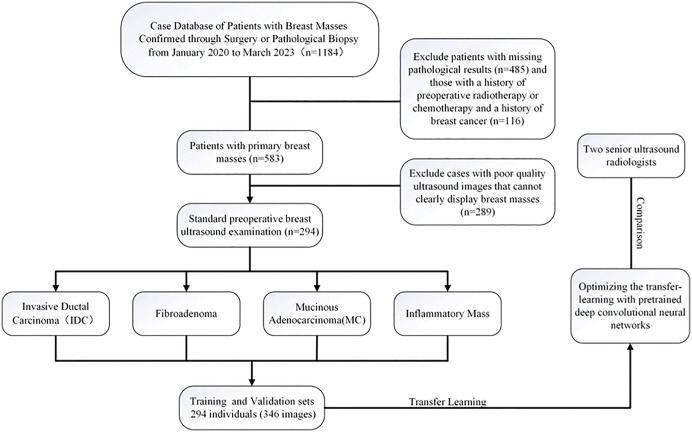
Illustrates the flowchart of the study process.

This study retrieved data from the hospital’s United-Imaging system for 1,184 patients who visited the Breast Surgery Department of the First Bethune Hospital of Jilin University between January 2020 and March 2023. Ultimately, 346 ultrasound images from 294 patients who underwent breast ultrasound examinations and had confirmed pathological results met the inclusion criteria. Among them, there were 75 cases of fibroadenoma, 67 cases of inflammatory mass, 99 cases of invasive carcinoma, and 53 cases of mucinous carcinoma. Most lesions were solitary, with a small portion being multiple. To prevent data leakage and ensure a fair evaluation, all eligible images were randomly split into training and test sets at a ratio of 8:2 after de-identification. Additionally, two senior ultrasound radiologists classified the images in the training set (Radiologist 1: 8 years of ultrasound diagnostic experience, Radiologist 2: 10 years of ultrasound diagnostic experience). Ultrasound images were acquired using the Canon Xario200 ultrasound diagnostic system, Canon Aplio300 ultrasound diagnostic system, Toshiba 790 ultrasound diagnostic system, and Philips HD9 ultrasound diagnostic system. Basic clinical data, including age, gender, pathological results, and ultrasound diagnostic reports, were obtained from the hospital’s electronic medical record system. Given the retrospective design of this study and the fact that all data were fully anonymized, the ethics committee approved a waiver of written informed consent. The patient information obtained during the study does not affect subsequent treatment nor compromise patient privacy.Fig 1 illustrates the flowchart of the study process.

Inclusion criteria: Definite pathological diagnosis results; ultrasound examination performed before biopsy or surgery; a single B-mode ultrasound image capable of fully displaying the boundary of the mass. Exclusion criteria: Uncertain pathological results; poor image quality; history of radiotherapy or chemotherapy; lack of ultrasound examination images.

### 2.2 Data preprocessing

The obtained breast mass ultrasound images were preprocessed by manually extracting the rectangular region containing the breast mass from the entire original ultrasound image to eliminate redundant information in the image. Finally, ultrasound images containing only other human tissue structures such as skin, subcutaneous fat, glandular tissue, chest muscles, intercostal muscles, etc., were obtained. In order to address issues such as insufficient feature learning, poor network performance, instability, and overfitting that may arise from small sample datasets during the training process, preprocessing and enhancement steps were applied to the image data, including image rotation, Gaussian blur, scaling, contrast, and brightness adjustments.All programs were executed in Python version Python 3.9.0.

### 2.3 Training and interpretation of deep learning models

Transfer learning, as a machine learning method, aims to apply knowledge or experience learned from one task to another related task [25]. The study selected transfer learning using pre-trained deep convolutional neural network models. During the model training phase, pre-training was conducted using the ImageNet dataset [26], Five deep learning models, namely ResNet18, ResNet50 [27], DenseNet121 [28], MobileNetV2 [29], and GoogLeNet [30], were used as base networks. The parameters of the convolutional layers were retained, and fine-tuning was performed on the classification layer parameters using the training set to classify breast mass ultrasound images into four types.

**Fig 2:**
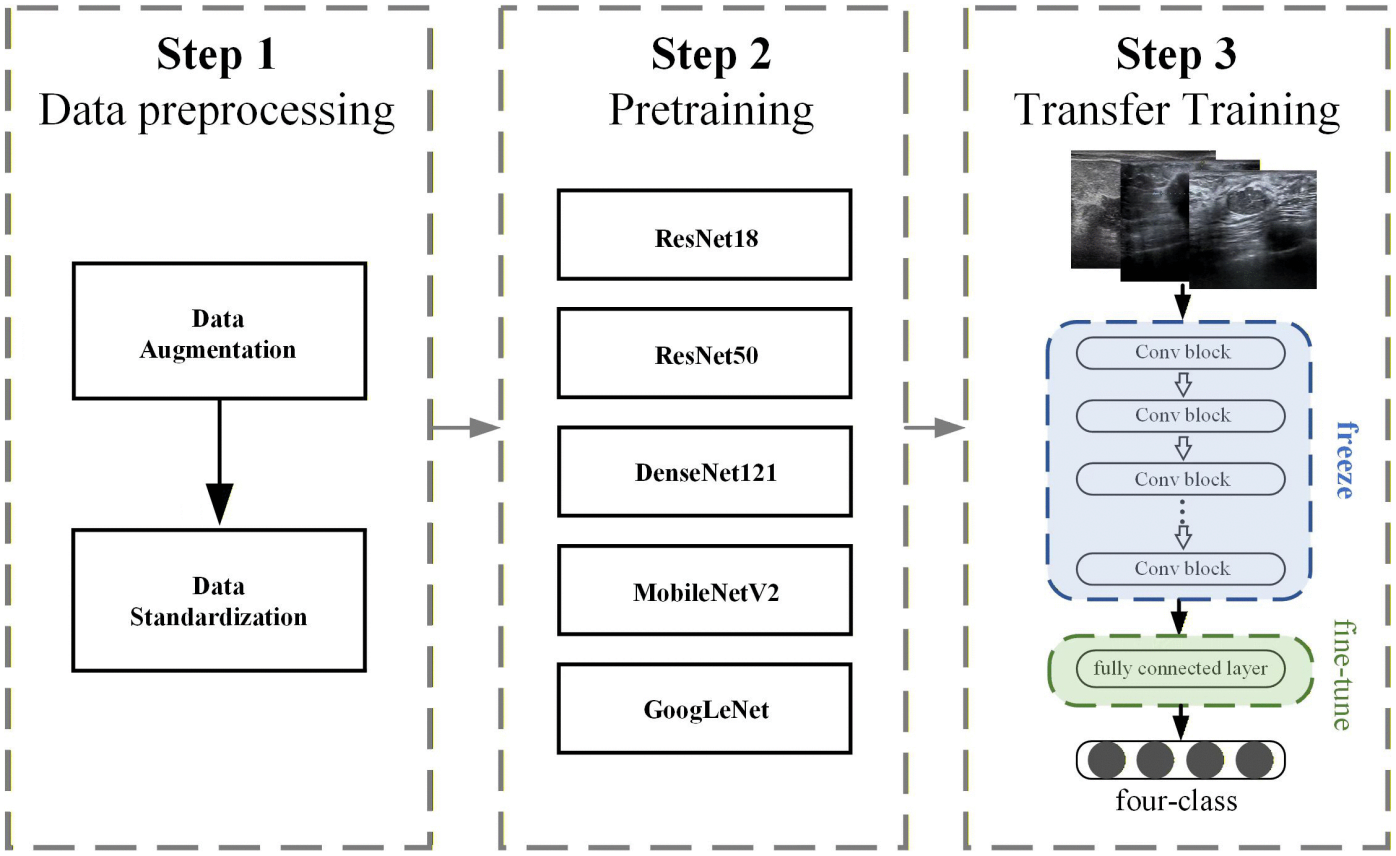
Overview of feature based transfer learning framework.

This study chose the ADAM optimizer as the optimization algorithm for the model and utilized it to update gradients and network parameters. Cross-entropy loss function was employed to measure the difference between model output and true labels. Regarding hyperparameter settings, the batch size (BS) was set to 32, the number of epochs was set to 50, and the initial learning rate (LR) was set to 0.001. Additionally, a learning rate decay strategy was adopted, halving the learning rate every 5 epochs to aid better convergence of the model. The Softmax function was used in the output layer of the model as a classification function to accomplish multi-class classification tasks. Fig 2 Overview of feature based transfer learning framework.

To better interpret the model’s prediction results, we employed class activation mapping (CAM) methods to generate heatmaps, visualizing the most indicative regions of interest in breast mass images as identified by the model [31,32]. All heat maps were generated using the OpenCV package (version 4.5.5).

### 2.4 Statistical analysis

The diagnostic performance of the five deep learning models was evaluated on the four-class breast mass classification task using standard metrics: accuracy, precision, recall (sensitivity), specificity, and F1-score. The models’ predictions were further visualized using confusion matrices and receiver operating characteristic (ROC) curves, with the area under the curve (AUC) calculated for each class. For benchmark comparison, the test set was independently classified by two senior ultrasound radiologists with 8 and 10 years of experience, respectively. McNemar’s test was employed to assess the statistical significance of the performance difference between each model and the radiologists’ consensus, with a p-value threshold of 0.01. These computations were carried out using standard scientific libraries in Python. The metrics are defined as follows:

Accuracy measures the overall proportion of correctly classified instances and is calculated as:


$$\text{Accuracy}=\frac{\text{True}\;\text{Positives}+\text{True}\;\text{Negatives}}{\text{Total}\;\text{Samples}}$$ (1)

Precision quantifies the proportion of correctly predicted positive instances among all instances predicted as positive:


$$\text{Precision}=\frac{\text{True}\;\text{Positives}}{\text{True}\;\text{Positives}+\text{False}\;\text{Positives}}\;$$ (2)

Recall (also known as Sensitivity) indicates the proportion of actual positive instances that are correctly identified:


$$\text{Recall}\;=\frac{\text{True}\;\text{Positives}}{\text{True}\;\text{Positives}+\text{False}\;\text{Negatives}}\;$$ (3)

Specificity assesses the model’s ability to correctly identify negative instances:


$$\text{Specificity}\;=\frac{\text{True}\;\text{Negatives}}{\text{True}\;\text{Negatives}+\text{False}\;\text{Positives}}$$ (4)

F1-score represents the harmonic mean of precision and recall, providing a single metric that balances both concerns:


$$\text{F1}\;\text{Score}\;=\frac{\text{2}\times \text{Precision}\times \text{Recall}}{\text{Precision}+\text{Recall}}$$ (5)

## 3. Results

### 3.1 Patient and ultrasound image characteristics

This study included 346 ultrasound images of breast masses from 294 patients with breast masses, including 99 cases of invasive ductal carcinoma, 75 cases of fibroadenoma, 67 cases of inflammatory masses, and 53 cases of mucinous carcinoma, as shown in Table 1. All ultrasound images were randomly allocated to the training set and testing set, with 278 breast mass images used to train the models and optimize their parameters, and the remaining 68 breast mass images used as an independent test set to validate the performance of the trained optimal models.

**Table 1:** Demographic data for 294 patients.

	Invasive ductal carcinoma(lDC)	Fibroadenoma	Inflammatory masses	Mucinous Careinomaf(MC)
Masses no. (%)	99	75	67	53
Images no. (%)	105	96	84	61
Age				
Mean (y)	52.01 ± 11.55	35.77 ± 10.50	40.83 ± 12.42	54.61 ± 12.50
Range (y)	31-79	15-68	16-69	32-87
Maximum diameter				
Mean (mm)	22.85 ± 11.03	17.79 ± 7.70	19.91 ± 9.29	24.53 ± 8.45
Range (mm)	7.2-58.2	4.8-44.2	4.7-43.8	7.4-42.4

### 3.2 Comparison of diagnostic performance between the five transfer learning models and radiologists

**Fig 3:**
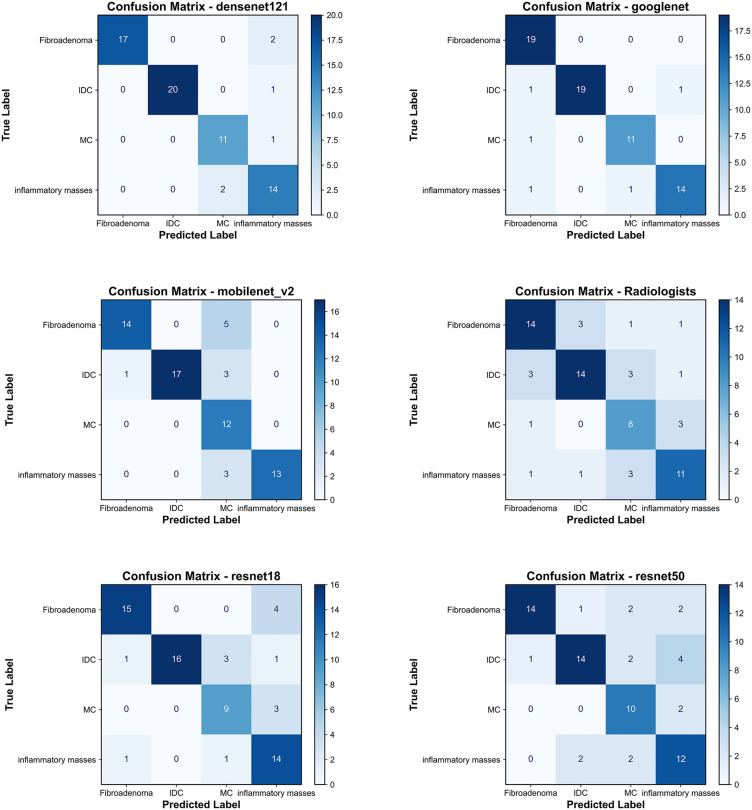
Confusion matrices of transfer learning models and radiologists on the test set.

Among the five pre-trained deep convolutional neural network models used in this study, DenseNet121 performed excellently in classifying the four categories. Additionally, the accuracy of the DenseNet121 model and senior radiologists for invasive ductal carcinoma, fibroadenoma, mucinous carcinoma, and inflammatory mass were 0.9853, 0.9706, 0.9559, 0.9118, and 0.8382, 0.8529, 0.8382, 0.8529, respectively. Fig 3 shows the classification confusion matrices of different models and senior ultrasound radiologists, detailing the numbers of true positive, false positive, true negative, and false negative results. Specific diagnostic performance metrics and results are listed in Table 2.

**Table 2:** Classification performance of transfer learning models and radiologists.

Models/ Radiologists	Class	Accuracy	Specificity	Precision	Recall	F1 Score
ResNet18	Fibroadenoma	0.9118	0.9592	0.8824	0.7895	0.8333
	lDC	0.9265	1.0000	1.0000	0.7619	0.8649
	MC	0.8971	0.9286	0.6923	0.7500	0.7200
	IM	0.8529	0.8462	0.6364	0.8750	0.7368
ResNet50	Fibroadenoma	0.9118	0.9796	0.9333	0.7368	0.8235
	lDC	0.8529	0.9362	0.8235	0.6667	0.7368
	MC	0.8824	0.8929	0.6250	0.8333	0.7143
	IM	0.8235	0.8462	0.6000	0.7500	0.6667
DenseNet121	Fibroadenoma	0.9706	1.0000	1.0000	0.8947	0.9444
	lDC	0.9853	1.0000	1.0000	0.9524	0.9756
	MC	0.9559	0.9643	0.8462	0.9167	0.8800
	IM	0.9118	0.9231	0.7778	0.8750	0.8235
GoogLeNet	Fibroadenoma	0.9559	0.9388	0.8636	1.0000	0.9268
	lDC	0.9706	1.0000	1.0000	0.9048	0.9500
	MC	0.9706	0.9821	0.9167	0.9167	0.9167
	IM	0.9559	0.9808	0.9333	0.8750	0.9032
MobileNetV2	Fibroadenoma	0.9118	0.9796	0.9333	0.7368	0.8235
	lDC	0.9412	1.0000	1.0000	0.8095	0.8947
	MC	0.8382	0.8036	0.5217	1.0000	0.6857
	IM	0.9559	1.0000	1.0000	0.8125	0.8966
Radiologists	Fibroadenoma	0.8529	0.8980	0.7368	0.7368	0.7368
	lDC	0.8382	0.9149	0.7778	0.6667	0.7179
	MC	0.8382	0.8750	0.5333	0.6667	0.5926
	IM	0.8529	0.9038	0.6875	0.6875	0.6875

Table 3 summarizes the diagnostic performance of the deep learning models in comparison to radiologists. The DenseNet121 and GoogLeNet architectures significantly outperformed the radiologists’ consensus (OA: 0.691), achieving overall accuracies of 0.912 and 0.926, respectively (McNemar’s test, p < 0.01). While all models attained higher overall accuracy than the radiologists, the performance advantage of ResNet18, ResNet50, and MobileNet-V2 was not statistically significant (p > 0.05).

**Table 3:** Performance comparison of transfer learning models versus radiologists in breast mass classification.

Model	OA	Fibroadenoma	IDC	MC	IM	McNemar p-value
ResNet18	0.794	0.789	0.762	0.750	0.875	0.0704
ResNet50	0.735	0.737	0.667	0.833	0.750	0.6625
DenseNet121	0.912	0.895	0.952	0.917	0.875	0.0007**
GoogLeNet	0.926	1.000	0.905	0.917	0.875	0.0004**
MobileNetV2	0.824	0.737	0.810	1.000	0.812	0.1096
Radiologists	0.691	0.737	0.667	0.667	0.688	–

Note: OA: Overall Accuracy. The p-values are from McNemar’s test comparing each model with radiologists. ** indicates p < 0.01.

**Fig 4:**
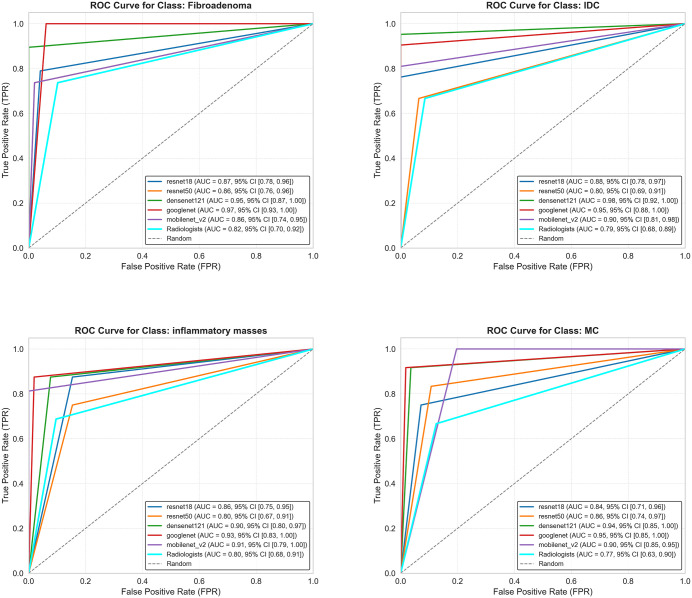
Receiver operating characteristic curves of transfer learning models and radiologists on the test set.

Fig 4 shows the ROC curves for different models and senior ultrasound radiologists on the test set for classifying different masses. The DenseNet121 model had the highest AUC value in classifying invasive ductal carcinoma (0.98, 95% CI: 0.917–1.000). The GoogLeNet model performed best in classifying fibroadenoma, inflammatory masses, and mucinous carcinoma, with AUC values of 0.97 (95% CI: 0.932–1.000), 0.93 (95% CI: 0.833–1.0000), and 0.95 (95% CI: 0.850–1.000), respectively. Senior radiologists had AUC values of 0.79 (95% CI: 0.683–0.792), 0.81 (95% CI: 0.702–0.919), 0.80 (95% CI: 0.676–0.912), and 0.77 (95% CI: 0.629–0.904) for classifying invasive ductal carcinoma, fibroadenoma, inflammatory masses, and mucinous carcinoma, respectively.

**Fig 5:**
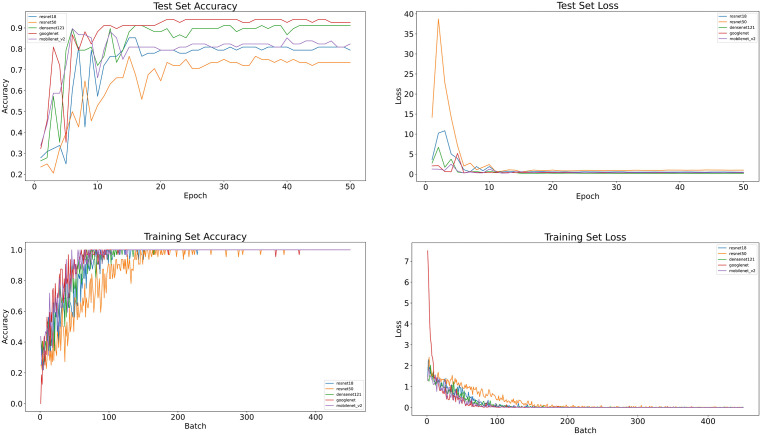
Training and test set curves for each model.

Fig 5 shows the training curves of the five pre-trained deep learning models. The ascending curves represent the accuracy on the training and validation sets, while the descending curves represent the loss on the training and test sets, indicating the degree of fit between the predictions and the true pathological labels. As the number of training epochs increases, the accuracy on both the training and test sets gradually rises and stabilizes, while the loss function curves for both the training and test sets continuously decrease. The similarity between the two loss curves indicates that the model does not exhibit significant overfitting.

**Fig 6:**
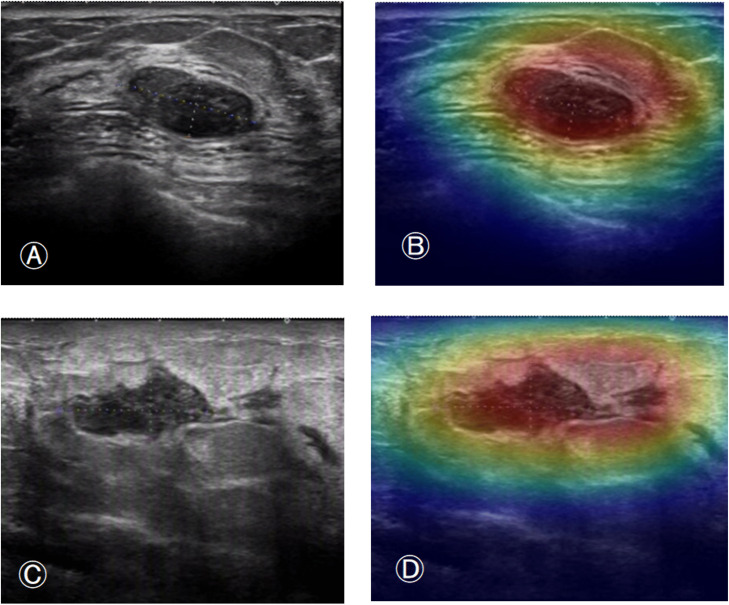
Figures A and C show B-mode ultrasound images of a fibroadenoma and an inflammatory masses, respectively, from the test set, and Figures C and D show the corresponding images after overlaying the thermograms using the pre-trained DenseNet121 model.

**Fig 7:**
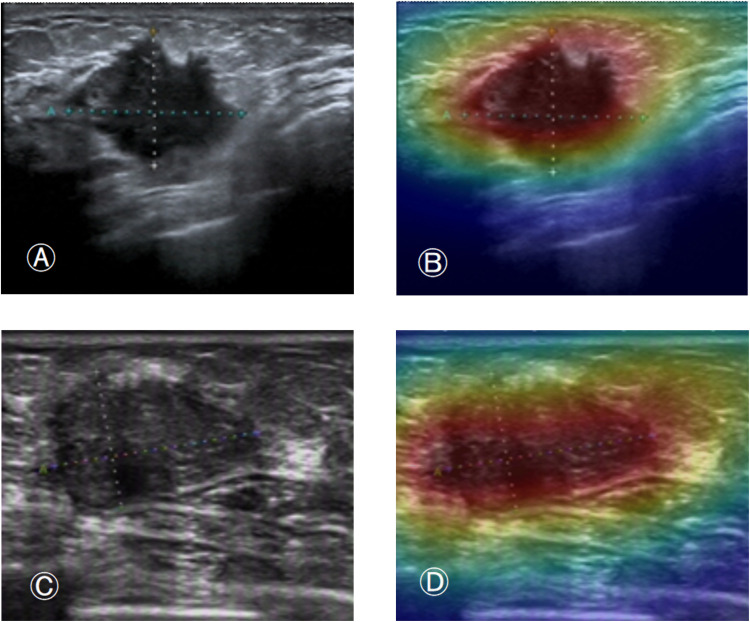
Figures A and C show the B-mode ultrasound images of invasive ductal carcinoma and mucinous carcinoma in the test set, respectively, and Figures C and D show the respective images after overlaying the thermograms using the pre-trained DenseNet121 model.

In Fig 6, images A and C show the ultrasound images of fibroadenoma and inflammatory mass in the test set, respectively, while images B and D show the corresponding images with heatmaps overlaid using the pre-trained DenseNet121 model. In Fig 7, images A and C show the ultrasound images of invasive ductal carcinoma and mucinous carcinoma in the test set, respectively, while images B and D show the corresponding images with heatmaps overlaid using the pre-trained DenseNet121 model. The model correctly predicted each of their true categories.

## 4. Discussion

This study demonstrated the efficacy of optimized transfer learning with pre-trained deep convolutional neural networks (DCNNs) for the four-category classification of breast tumors in ultrasound images. The employed models—ResNet18, ResNet50, DenseNet121, GoogLeNet, and MobileNetV2—all achieved satisfactory classification outcomes, particularly the DenseNet121 model, which produced commendable results on the test set. The areas under the receiver operating characteristic curve (AUC) for invasive ductal carcinoma, fibroadenoma, mucinous carcinoma, and inflammatory breast tumors were 0.976 (95% CI: 0.917, 1.000), 0.947 (95% CI: 0.870, 1.000), 0.940 (95% CI: 0.845, 1.000), and 0.899 (95% CI: 0.802, 0.974), respectively, surpassing the performance of two experienced sonographers. Notably, McNemar’s test revealed statistically significant differences between radiologists and both GoogleNet (p < 0.001) and DenseNet121 (p = 0.001), indicating that these models performed significantly better than human experts. In contrast, ResNet18 (p = 0.070), ResNet50 (p = 0.663), and MobileNet-V2 (p = 0.110) showed no statistically significant difference from radiologists, suggesting comparable performance levels.

From Table 2, we observe that although all five models achieved satisfactory prediction results on the test set, there were slight differences in classification among the different categories. Overall, DenseNet121 and GoogLeNet performed exceptionally well across various categories. In clinical practice, for the ultrasound diagnosis of breast tumors, we aim to find the best method to minimize the misdiagnosis rate of benign tumors and the missed diagnosis rate of malignant tumors.To this end, we analyzed diagnostic errors and identified specific patterns. A key finding was that inflammatory masses were most frequently misclassified as malignant tumors, particularly mucinous carcinoma, which could lead to unnecessary biopsies. Conversely, the majority of missed malignancies were also mucinous carcinomas, as their sonographic features overlap with benign conditions. Considering the overall performance, DenseNet121 appears to be a more reliable choice for clinical application in the classification and prediction of breast tumor ultrasound images.

Figs 5 and 6 shows the heatmaps generated by the pre-trained DenseNet121 model using the CAM class activation mapping method. These heatmaps illustrate the decision basis for the model’s classification of breast tumors. In these images, the model correctly predicted the true category of each image. Notably, the model’s attention is not limited to the tumor region but also includes parts of the tumor margins. This indicates that these regions also contribute value to the model’s prediction process. It also suggests that the model comprehensively understands the image and makes accurate classification predictions based on the different features of each region.

Despite the achievements of this study, several limitations remain. First, the retrospective design and relatively small dataset may pose risks of overfitting and affect the model’s generalizability. To mitigate overfitting, we implemented a rigorous random assignment of images during data preprocessing and employed extensive data augmentation techniques. More importantly, the use of transfer learning with pre-trained models, which leverages features learned from large-scale natural image datasets (e.g., ImageNet), significantly enhances feature extraction capability and robustness when fine-tuned on smaller medical datasets. The consistently superior performance of our models, particularly DenseNet121, strongly suggests that the models have effectively learned generalizable pathological features rather than merely memorizing dataset-specific noise. However, further validation is required to assess model performance on external datasets acquired from different institutions and imaging equipment.

Second, since the examinations were conducted by multiple radiologists, some variations in image quality and acquisition parameters are inevitable, which, while reflecting real-world clinical practice, may introduce confounding factors. Third, the selection of pre-trained deep convolutional neural network models, though encompassing diverse architectures (e.g., residual networks, densely connected networks), is not exhaustive, and other state-of-the-art architectures could be explored.

Lastly, the model’s classification performance for rare or atypical cases requires further validation. The clinical impact of misclassification, particularly false negatives and false positives, must be carefully considered. A false negative (e.g., misclassifying a malignant mass as benign) could lead to delayed diagnosis and treatment, with serious consequences for patient outcomes. Conversely, a false positive (e.g., classifying a benign mass as malignant) may cause unnecessary patient anxiety, follow-up examinations, or invasive biopsies, increasing healthcare costs and patient burden. Therefore, any clinical deployment must prioritize high sensitivity, especially for malignant categories.

For future clinical implementation, the proposed model is best positioned not as a replacement, but as a decision support tool integrated into the radiology workflow. It could function as a pre-screening system to flag suspicious cases for prioritized radiologist review, thereby improving diagnostic efficiency. Alternatively, it could serve as a “second reader” to highlight potential discrepancies and reduce perceptual errors. To realize this vision, prospective, multi-center studies are essential to validate the model’s efficacy in a live clinical setting and to understand its impact on radiologists’ diagnostic accuracy and workflow. Furthermore, generalization capability must be enhanced by training on larger, more diverse, multi-institutional datasets and by refining the model architecture to improve its sensitivity to atypical and rare lesions.

## 5. Conclusion

In conclusion, this study successfully explored the use of pre-trained deep convolutional neural networks for optimized transfer learning to achieve four-class prediction of breast masses in ultrasound images. This method could become an effective tool in clinical practice for early screening of different types of breast masses, supporting radiologists in better formulating treatment strategies, increasing the chances of early detection of breast cancer, and positively impacting the health of female patients.
